# Ultrasound-Guided Suprainguinal Fascia Iliaca Compartment Block in Patients Undergoing Hip Surgery: A Systematic Review and Meta-Analysis of Randomized Controlled Trials

**DOI:** 10.7759/cureus.70147

**Published:** 2024-09-25

**Authors:** Yonghan Li, Chloe Soo Suan Chai, Chin Koon Alex Koh, Chi Ho Chan

**Affiliations:** 1 Department of Anesthesiology, Sengkang General Hospital, Singapore, SGP; 2 Department of Anesthesiology, Singapore General Hospital, Singapore, SGP

**Keywords:** arthroscopic hip surgery, hip fracture, regional anesthesia, suprainguinal, total hip replacement

## Abstract

The use of fascia iliaca compartment block (FICB) has been widely encouraged for hip surgery; however, meta-analyses showed mixed results in terms of its efficacy in reduction in analgesic consumption and pain score. These meta-analyses included all forms of FICB approaches, which may diminish the effect size of the therapy. Suprainguinal FICB (s-FICB) has been shown to be superior to other FICB approaches including the ultrasound-guided infrainguinal approach and the landmark approach. This systematic review and meta-analysis aim to compare opioid consumption, pain score, and complications after s-FICB to control for patients undergoing hip surgery.

The study protocol was registered with the International Prospective Register of Systematic Reviews (PROSPERO) (registration number CRD42023460377). We performed a systematic literature search in Medical Literature Analysis and Retrieval System Online (MEDLINE), Embase, Scopus, and Cochrane Central Register of Controlled Trials (CENTRAL) electronic databases from inception to 16 August 2023 to identify randomized controlled trials (RCTs) that evaluated the efficacy of s-FICB versus control for patients undergoing hip surgery. Data were independently extracted by two reviewers, and disagreements were resolved by consensus or by discussion with a third investigator. The primary outcome is the 24-hour oral morphine equivalent daily dose (oMMED). The secondary outcome includes oMMED at different timepoints, and pain score. The Cochrane risk of bias tool (Cochrane, London, England) was used to assess the risk of bias. The certainty of evidence was assessed via the Grading of Recommendations Assessment, Development and Evaluation (GRADE) approach. Data were synthesized using a random-effects model. Trial sequence analysis is performed on opioid consumption 24 hours post operation.

Eleven randomized controlled trials were included. Arthroscopic hip surgery was performed in three studies involving 222 patients, hip and femur fracture surgeries were performed in three studies involving 149 patients, and total hip arthroplasty was performed in five studies involving 483 patients. In studies involving arthroscopic hip surgery, s-FICB did not improve intra-operative and post-operative opioid consumption and post-operative pain score. In studies involving hip and femur fracture surgeries, s-FICB was associated with a non-significant difference in opioid consumption at 24 hours after surgery and post-operative pain score at 12 hours and 24 hours after surgery. However, the result of the trial sequential analysis (TSA) was not definitive, indicating that additional research is necessary to draw conclusive outcomes. In studies involving total hip arthroplasty, s-FICB was associated with a significant reduction in post-operative opioid consumption at 24 and 48 hours with conclusive results in trial sequential analysis.

In conclusion, s-FICB is superior to placebo for patients undergoing total hip arthroplasty. For patients undergoing arthroscopic hip surgery, s-FICB is unlikely to be beneficial. With regard to hip fracture surgery, additional research is necessary to draw conclusive outcomes.

## Introduction and background

Fascia iliaca compartment block (FICB) provides the denervation of the femoral nerve, obturator nerve, and lateral femoral cutaneous nerve, thus providing effective analgesia coverage of the hip joint, femur osteotome, and the site of incision during hip surgery [1]. In addition, FICB may help facilitate positioning for spinal anesthesia in patients with hip fractures, potentially increasing spinal anesthesia success [2,3]. Traditionally, FICB is performed under the landmark-guided approach described by Dalens et al. until the utilization of ultrasound-guided methodology gained widespread acceptance [4]. The ultrasound-guided infrainguinal FICB (i-FICB) was first described by Dolan et al. and has been shown to provide a more effective sensory and motor nerve block of the three nerves when compared to the landmark approach [5]. Subsequently, an ultrasound-guided suprainguinal FICB (s-FICB) approach was described by Hebbard et al. in 2011 in a cadaver study [6]. s-FICB significantly improved the successful denervation of the obturator nerve [7] and has been shown to provide better analgesia for hip fracture analgesia [8] and hip surgery [9] when compared to i-FICB.

Multiple meta-analyses have been published on FICB in patients undergoing hip surgery with conflicting results. For total hip arthroplasty, while most meta-analyses demonstrated a reduction in opioid consumption [10,11], a reduction in pain score up to 12 hours [10-12] and 24 hours [12], and a reduction in nausea [10-12], a more recent meta-analysis found no difference in opioid consumption and pain score [13]. Moreover, the impact of FICB on the length of stay is not consistent [11,12]. Mortality benefit has yet to be demonstrated [12]. For hip fracture surgery, Hong and Ma demonstrated a reduction in opioid consumption, a reduction in pain score for up to 48 hours, and a reduction in nausea with single-shot FICB [14]; however, Baker et al. found no difference in morphine consumption after single-shot FICB, while morphine consumption reduction was observed when FICB catheters were used [15]. Arguably, these meta-analyses have included FICB performed under a variety of techniques including the landmark approach [11-15] and the infrainguinal approach [10-15]. As mentioned above, landmark FICB and i-FICB may be inferior to s-FICB and, as a result, diminish the magnitude of the effect of these post-operative outcomes.

This systematic review and meta-analysis aim to compare opioid consumption, pain score, and complications after s-FICB to control for patients undergoing hip surgery. We hypothesize that patients undergoing hip surgery with s-FICB would have a significant reduction in pain score and opioid consumption and opioid-related side effects. We also aim to identify gaps in the existing evidence and provide recommendations for further studies needed.

## Review

Methods

This systematic review and meta-analysis was written in accordance with the Preferred Reporting Items for Systematic Reviews and Meta-Analyses (PRISMA) guidelines [16]. The study protocol was registered with the International Prospective Register of Systematic Reviews (PROSPERO) (registration number CRD42023460377).

Search Strategy

We performed a systematic literature search in Medical Literature Analysis and Retrieval System Online (MEDLINE), Embase, Scopus, and Cochrane Central Register of Controlled Trials (CENTRAL) electronic databases from inception to 16 August 2023. The search keywords were as follows: (hip fracture OR hip surgery OR femur fracture) AND (Fascia iliaca block OR Fascia iliaca nerve block OR Fascia iliaca compartment block OR Fascia iliaca compartment nerve block OR Fascia iliac block OR Fascia iliac nerve block OR Fascia iliac compartment block OR Fascia iliac compartment nerve block OR Fascia-iliaca block OR Fascia-iliaca nerve block OR Fascia-iliaca compartment block OR Fascia-iliaca compartment nerve block OR Fascia-iliac block OR Fascia-iliac nerve block OR Fascia-iliac compartment block OR Fascia-iliac compartment nerve block OR FICB OR FIC OR FIB). Only clinical trials were included in the search. We also checked the references of previous meta-analyses to identify other potentially eligible trials.

Study Selection

**Figure 1:**
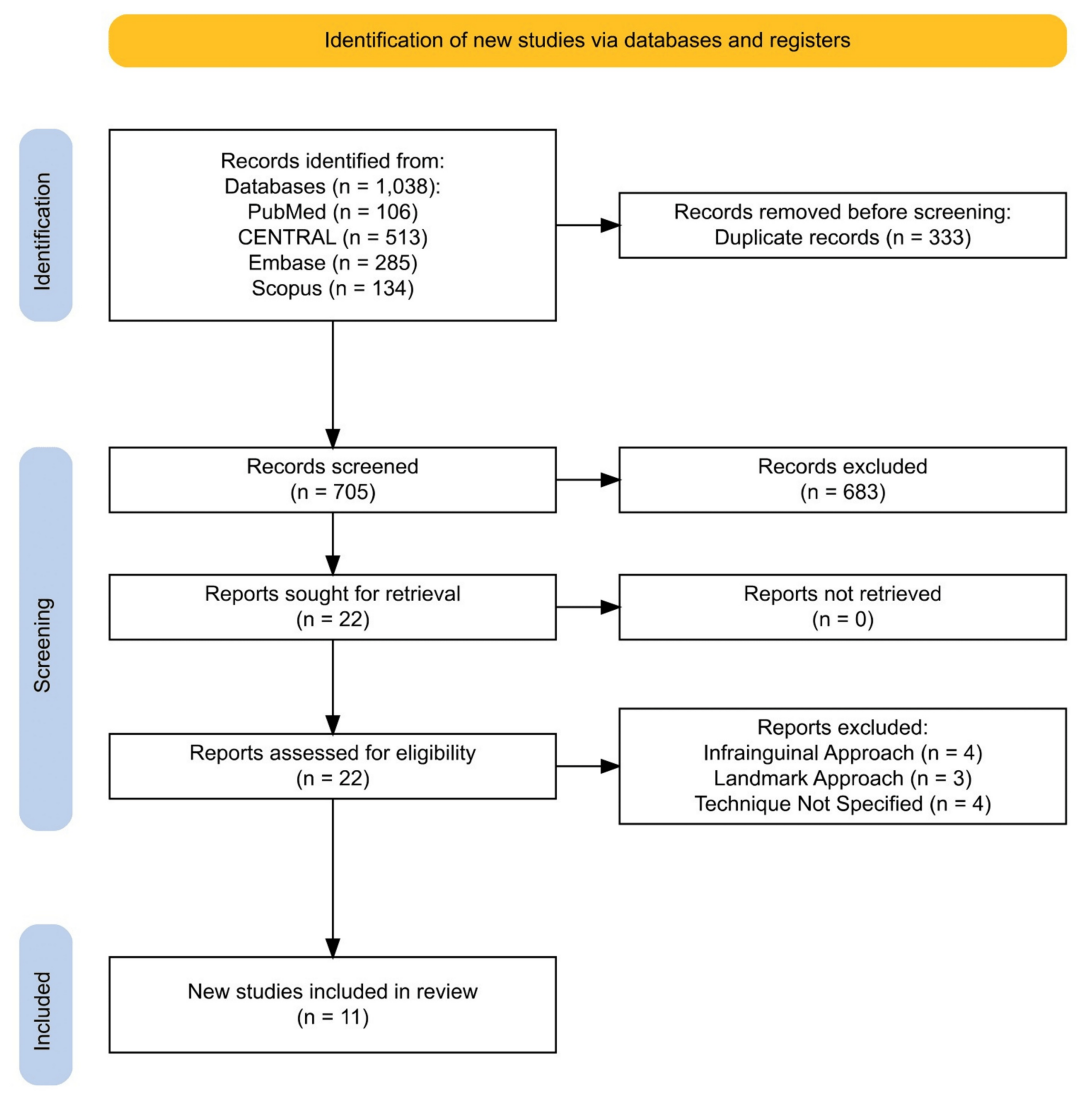
PRISMA flow diagram representing the search and selection of studies comparing s-FICB to control. PRISMA, Preferred Reporting Items for Systematic Reviews and Meta-Analyses; s-FICB, suprainguinal fascia iliaca compartment block; CENTRAL, Cochrane Central Register of Controlled Trials

We included studies of patients undergoing hip surgery who received ultrasound-guided s-FICB perioperatively versus the control cohort who did not receive the block. Only a human randomized controlled trial (RCT) was included in this meta-analysis. No language restrictions were applied. After removing duplicated studies, the titles and abstracts of the studies were screened by two independent investigators for eligibility. If there was doubt during title and abstract screening, the full-text article was obtained and appraised. Controversies, if any, were settled by consensus or discussion with a third investigator. A flow diagram is presented in Figure 1.

Quality Assessment

**Figure 2:**
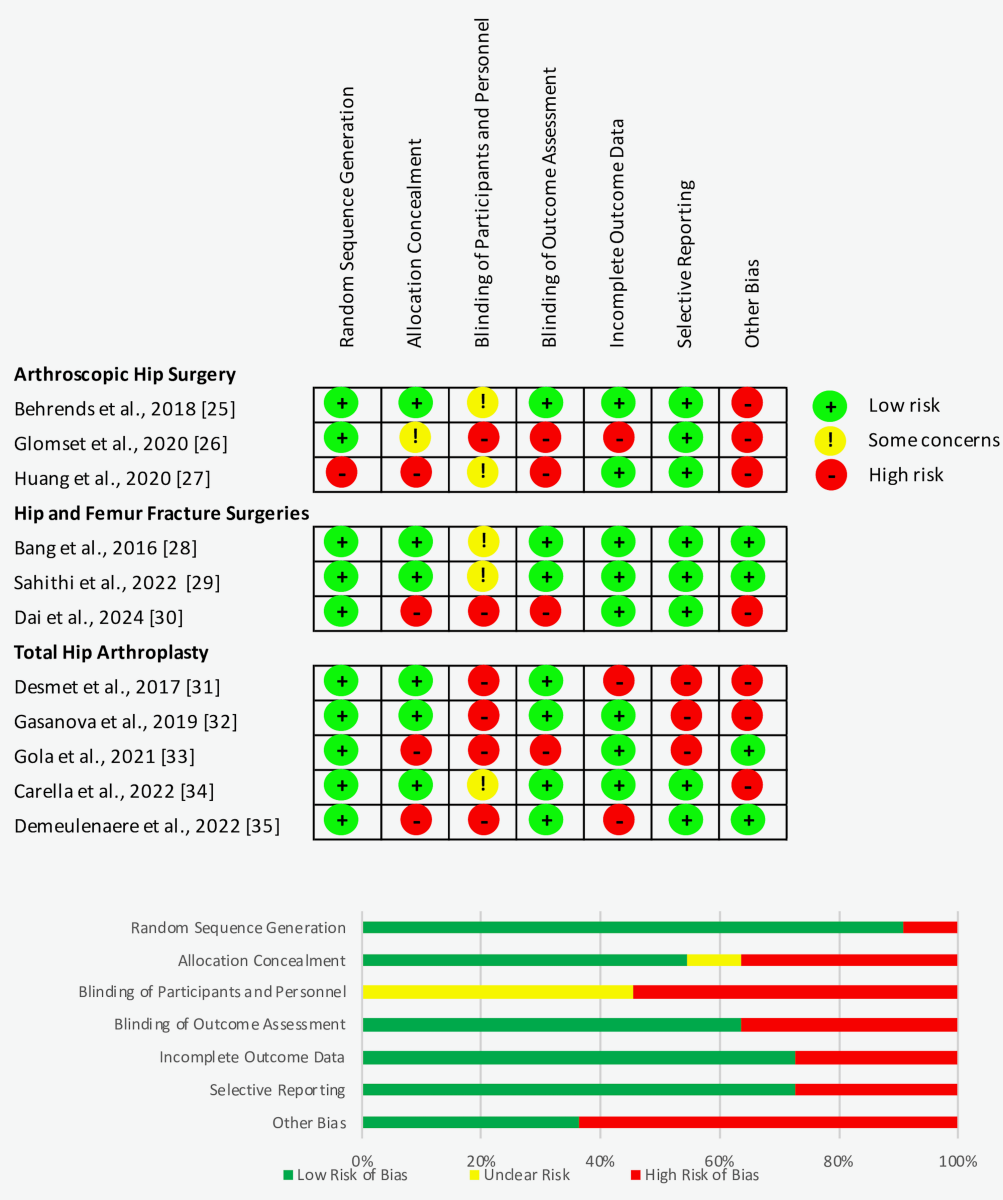
Risk of bias summary based on the Cochrane Collaboration “risk of bias” tool.

The quality of each individual study was assessed objectively using the Cochrane Collaboration “risk of bias” tool (Cochrane, London, England). The assessment tool assesses for seven potential risks of bias including (i) details of the randomization method, (ii) allocation concealment, (iii) the blinding of participants and personnel, (iv) blind outcome assessment, (v) incomplete outcome data, (vi) selective outcome reporting, and (vii) other sources of bias. Each aspect is graded as (+) low risk of bias, (-) high risk of bias, and (?) unclear risk of bias. The quality assessment of individual studies in this meta-analysis was reviewed by two independent investigators and presented in Figure 2. The Grading of Recommendations Assessment, Development and Evaluation (GRADE) assessment was used to assess for the certainty of evidence [17].

Data Extraction

The data extraction from the eligible articles was performed independently by two investigators, and disagreements were resolved by consensus or by discussion with a third investigator. The extracted data include publication year, the size of each group, mean age, gender, surgery performed, interventional details for both intervention and control group (such as drug, dose, and the timing of the administration of block), intra-operative anesthesia technique, post-operative pain management plans, outcomes, and risk of bias. Opioid consumptions were converted to oral morphine equivalent daily dose (oMMED) using the Australian and New Zealand College of Anaesthetists Faculty of Pain Medicine opioid equivalence dose [18]. Pain scores are converted to a 100-point visual analog scale (VAS) score. If outcomes were presented only on a graph or bar chart and numeric values were not available, the outcome values were estimated from the graph or bar plot.

Missing Data

Data described as the median and range were translated into mean and standard deviation (SD) [19]. If the data were published as a figure without numerical values, the mean, median, range, or SD was estimated using the scale on the figure. If the data do not contain SD or range, an attempt was made to contact the corresponding authors of the respective studies; they were contacted for the missing data. Otherwise, the median value of SD from other studies of the same comparison was used to substitute for the missing SD [20].

Statistical Analysis

For continuous variables, we used the inverse variance weighting method and presented them as mean difference (MD) with a 95% confidence interval (CI). Data published as minimum, maximum, quartile ranges, median, and standard error with confidence intervals were converted to mean and standard deviation for meta-analysis [21-24]. Missing standard deviations are imputed using standard deviations from similar studies [20]. The analysis utilized a random-effects model, and the I^2 ^statistic was used to estimate the heterogeneity among the studies. Heterogeneity is deemed significant for values exceeding 50%. Statistically significant differences were indicated by P values of less than 0.05. Data were collected and analyzed using Review Manager (RevMan) version 5.4.1 (Cochrane, London, England). The risk of bias in individual studies was assessed using the Cochrane risk of bias tool.

We conducted a trial sequential analysis (TSA) of the included studies for opioid consumption at 24 hours. We used TSA software (version 0.9.5.10 β, Copenhagen Trial Unit, Copenhagen, Denmark) to perform this analysis. The TSA boundaries were constructed based on the O’Brien-Fleming alpha spending function. The Biggerstaff-Tweedie (BT) model was used due to the small number of studies and expected high heterogeneity. We also analyzed the results using the DerSimonian-Laird (DL) and Sidik-Jonkman (SJ) methods to observe any difference in results when different statistical models were used. The threshold for type 1 error was set at 5% and statistical power at 80%, and the estimated variance and heterogeneity were set from those present in the included trials.

Results

The search of the literature in different databases identified a total of 1,038 articles, of which 759 articles were included for screening after duplications from each database were excluded. After screening of the titles and abstracts, a full text of 22 articles was assessed for eligibility. A total of 11 RCTs met our inclusion criteria and were reviewed in our meta-analysis: arthroscopic hip surgery performed in three studies involving 222 patients [25-27], hip and femur fracture surgeries performed in three studies involving 149 patients [28-30], and total hip arthroplasty performed in five studies involving 483 patients [31-35]. A PRISMA flow diagram of the literature search is provided in Figure 1. The characteristics and demographics of the included studies are presented in Table 1. The risk of bias summary and risk of bias graph are shown in Figure 2. The GRADE assessment is presented in Table 2.

**Table 1:** Characteristic of the included studies. s-FICB, suprainguinal fascia iliaca compartmental block; PO, per os; IV, intravenous; PCA, patient-controlled analgesia; CR, controlled release; PACU, post-anesthesia care unit; NRS, numerical rating score; VAS, visual analog scale; BD, twice a day; Q6h, every six hours; Q12h, every 12 hours; Q3h, every three hours; Q8h, every eight hours; Q4h, every four hours; OD, once a day; OM, every morning; IM, intramuscular

	Intervention	Control	Sample size (mean age, year)		Intra-operative management	Perioperative adjunct therapy
			Intervention	Control		
Arthroscopic hip surgery						
Behrends et al. (2018) [25]	Pre-operative ultrasound-guided s-FICB, ropivacaine 0.2% 40 mL	Sham block, normal saline 0.9% 40 mL	38 (35)	40 (32)	General anesthesia, IV fentanyl as required. Intra-articular ropivacaine 0.2% 10 mL at the end of surgery	IV fentanyl and IV hydromorphone as needed to achieve an NRS ≤ 4 in PACU. Discharged with PO hydrocodone, PO acetaminophen, or PO oxycodone
Glomset et al. (2020) [26]​	Pre-operative ultrasound-guided s-FICB, ropivacaine 0.35% 3 mg/kg (up to 60 mL) with adrenaline 1:400,000 and clonidine 100 μg	Intra-articular injection of ropivacaine 0.5% 20 mL	41 (40.6)	43 (36.8)	General anesthesia, premedication with PO paracetamol 15 mg/kg, PO celecoxib 400 mg, PO pregabalin 75 mg, PO oxycontin 10 mg, and PO midazolam 1-5 mg. IV fentanyl as required.	IV hydromorphone 0.2-0.5 mg (with a maximum dose of 4 mg) in PACU
Huang et al. (2020) [27]	Pre-operative ultrasound-guided s-FICB, ropivacaine 0.35% 35-40 mL	No block	27 (42.4)	33 (41.4)	General anesthesia, premedication with PO pregabalin 150 mg and PO celecoxib 200 mg. Intra-operatively, IV paracetamol 1 g and other analgesia as required. Intra-articular plain bupivacaine 0.5% 10 mL with morphine 10 mg given via arthroscopic ports	IV fentanyl/oxycodone as required in PACU. Discharged with oxycodone as required and PO diazepam 5-10 mg for muscle spasm
Hip and femur fracture surgeries						
Bang et al. (2016) [28]	Post-operative ultrasound-guided s-FICB, ropivacaine 0.2% 40 mL with adrenaline 1:200,000	No block	11 (81)	10 (82)	Spinal anesthesia, hyperbaric bupivacaine 0.5% 2 mL with IV ketorolac 30 mg at the end of surgery	IV tramadol 25 mg as rescue analgesia in PACU or if the pain was not controlled by PACU. PO celecoxib 200 mg BD and PCA fentanyl at bolus dose 0.5 m μg/kg, a lockout of seven minutes, the four-hour limit of 4 μg/kg
Sahithi et al. (2022) [29]	Pre-operative ultrasound-guided s-FICB, ropivacaine 0.5% 30 mL	No block	33 (52)	33 (51)	Spinal anesthesia, hyperbaric bupivacaine 0.5% with fentanyl 25 μg (volume not documented)	IV paracetamol 1 g if VAS > 3, IV tramadol 100 mg if VAS > 6, and IV fentanyl 1 μg/kg If VAS > 6 after paracetamol and tramadol
Dai et al. (2024) [30]​	Post-operative ultrasound-guided s-FICB, ropivacaine 0.33% 30mL	No block	31 (76)	31 (74)	General anesthesia, IV sufentanil (0.3-0.5 μg/kg), IV lidocaine (1-2 mg/kg), and IV remifentanil titrated to response	PO flurbiprofen 1-2 mg/kg and PO tramadol
Total hip arthroplasty						
Desmet et al. (2017) [31]​	Pre-operative ultrasound-guided s-FICB, ropivacaine 0.5% 40 mL	No block	42 (60.4)	43 (66.5)	General anesthesia, IV paracetamol 1 g, IV diclofenac 75 mg, and IV sufentanil boluses as required	IV morphine in PACU as required. PCA morphine 1 mg bolus, a lockout of five minutes with four hours of 20 mg, IV paracetamol 1 g Q6h, and IV diclofenac 75 mg Q12h
Gasanova et al. (2019) [32]​​​​​​	Post-operative ultrasound-guided s-FICB, ropivacaine 0.5% 60 mL with adrenaline 1:400,000 and clonidine 100 μg	Periarticular injection of 60 mL mixture of ropivacaine 300 mg and adrenaline 150 μg	30 (56.2)	30 (59)	General anesthesia, premedication with PO gabapentin 600 mg and PO oxycodone CR 10 mg. Intra-operatively, IV paracetamol 1 g, IV ketorolac 30 mg, and IV fentanyl boluses as required	IV hydromorphone 0.1-0.2 mg as required in PACU if VAS > 4, 0.2-0.4 mg Q3h for post-operative day 1 as required. PO meloxicam 15 mg OD, PO gabapentin 300 mg Q8h, PO oxycodone CR 10 mg ON, and PO acetaminophen 1 g Q8h for post-operative day 1 and then changed to PO hydrocodone/acetaminophen 10 mg/325 mg Q4h as required
Gola et al. (2021) [33]	Post-operative ultrasound-guided s-FICB, ropivacaine 0.375% 40 mL with adrenaline 1:200,000	No block	50 (65)	50 (65)	Spinal anesthesia, 1.7-2.2 mL of hyperbaric bupivacaine 0.5% and premedication with PO paracetamol 500 mg, PO metamizole 500 mg, and PO pregabalin 75 mg	PCA oxycodone 1 mg bolus, a lockout of 10 minutes, and PO Oxycodone 10 mg Q12h for post-operative day 1. IV paracetamol 1 g Q6h and IV metamizole 1 g Q6h on post-operative day 1 and then changed to PO paracetamol 1 g Q6h and PO metamizole 1 g Q6h. PO pregabalin 75 mg OM
Carella et al. (2022) [34]​	Pre-operative ultrasound-guided s-FICB, ropivacaine 0.375% 40 mL	No block	43 (70)	43 (74)	Spinal anesthesia, 2 mL of isobaric bupivacaine 0.5% with sulfentanil 0.2 mL	PCA morphine 1 mg bolus, lockout of five minutes with four hours of 20 mg
Demeulenaere et al. (2022) [35]​	Post-operative ultrasound-guided s-FICB, ropivacaine 0.2% 40 mL	Control group 1: local infiltration of 150 mL of mixture of ropivacaine 300 mg, ketorolac 30 mg, and adrenaline 1:100,000. Control group 2: no block	49 (68)	Control group 1, 50 (68); control group 2, 53 (67)	Spinal anesthesia, 2.5 mL of hyperbaric bupivacaine 0.5%	IV or PO acetaminophen 1 g Q6h (or reduced to 3 g per 24 hours if with age ≥ 75 years old, weight < 50 kg, liver failure, and chronic ethyl abuse), IM or PO diclofenac 75 mg Q12h, IV tramadol 100 mg, or PO tramadol 50 mg (or reduced to 300 mg per 24 hours if with age ≥ 75 years old, 100 mg per 24 hour if with liver failure, or 200 mg per 24 hours if with kidney failure) as required. IM piritramide 10 mg Q6h as required

**Table 2:** Grading of Recommendations Assessment, Development and Evaluation (GRADE) assessment. ⨁⨁⨁⨁, high certainty of evidence; ⨁⨁⨁⊝, moderate certainty of evidence; ⨁⨁⊝⊝, low certainty of evidence ^a^Small sample bias may exist ^b^Risk of bias MD, mean difference; CI, confidence interval; RCT, randomized controlled trial; PACU, post-anesthesia care unit; VAS, visual analog scale

	Number of studies (design)	Risk of bias	Inconsistency	Indirectness	Imprecision	Other considerations	Effect	Certainty	Importance
Arthroscopic hip surgery									
Opioid consumption intra-operative	2 studies (RCT)	Not serious	Not serious	Not serious	Not serious	Not serious	MD, -2.94; 95% CI, -6.75 to 0.88; p = 0.13; I^2^ = 18%	⨁⨁⨁⊝^a^	Critical
Opioid consumption at PACU	3 studies (RCT)	Serious	Not serious	Not serious	Not serious	Not serious	MD, -0.73; 95% CI, -5.50 to 4.03; p = 0.76; I^2^ = 21%	⨁⨁⊝⊝^a,b^	Critical
Opioid consumption at 24 hours post operation	2 studies (RCT)	Not serious	Not serious	Not serious	Not serious	Not serious	MD, -0.79; 95% CI, -7.94 to 6.36; p = 0.83; I^2^ = 6%	⨁⨁⨁⊝^a^	Critical
VAS score at PACU	3 studies (RCT)	Serious	Not serious	Not serious	Not serious	Not serious	MD, 0.02; 95% CI, -0.36 to 0.41; p = 0.90; I^2^ = 0%	⨁⨁⊝⊝^a,b^	Critical
Hip and femur fracture surgeries									
Opioid consumption at 24 hours post operation	3 studies (RCT)	Not serious	Not serious	Not serious	Not serious	Not serious	MD, -7.78; 95% CI, -16.73 to 1.17; p = 0.09; I^2^ = 70%	⨁⨁⨁⊝^a^	Critical
VAS score at 12 hours post operation	2 studies (RCT)	Not serious	Not serious	Not serious	Not serious	Not serious	MD, -0.02; 95% CI, -0.19 to 0.24; p = 0.83; I^2^ = 0%	⨁⨁⨁⊝^a^	Critical
VAS score at 24 hours post operation	3 studies (RCT)	Not serious	Not serious	Not serious	Not serious	Not serious	MD, -0.41; 95% CI, -1.06 to 0.25; p = 0.22; I^2^ = 81%	⨁⨁⨁⊝^a^	Critical
Total hip arthroplasty									
Opioid consumption at 24 hours post operation	5 studies (RCT)	Not serious	Not serious	Not serious	Not serious	Not serious	MD, -25.94; 95% CI, -38.10 to -13.78; p < 0.00001; I^2^ = 93%	⨁⨁⨁⨁	Critical
Opioid consumption at 48 hours post operation	4 studies (RCT)	Not serious	Not serious	Not serious	Not serious	Not serious	MD, -39.02; 95% CI, -59.48 to -18.57; p < 0.0001; I^2^ = 86%	⨁⨁⨁⨁	Critical
VAS score at PACU	4 studies (RCT)	Not serious	Not serious	Not serious	Not serious	Not serious	MD, -1.37; 95% CI, -2.46 to -0.28; p = 0.01; I^2^ = 71%	⨁⨁⨁⨁	Critical
VAS score at 12 hours post operation	4 studies (RCT)	Not serious	Not serious	Not serious	Not serious	Not serious	MD, -0.24; 95% CI, -0.65 to 0.17; p = 0.37; I^2^ = 4%	⨁⨁⨁⨁	Critical
VAS score at 24 hours post operation	4 studies (RCT)	Not serious	Not serious	Not serious	Not serious	Not serious	MD, -0.16; 95% CI, -0.70 to 0.37; p = 0.11; I^2^ = 50%	⨁⨁⨁⨁	Critical
VAS score at 48 hours post operation	4 studies (RCT)	Not serious	Not serious	Not serious	Not serious	Not serious	MD, -0.31; 95% CI, -0.61 to -0.02; p = 0.56; I^2^ = 0%	⨁⨁⨁⨁	Critical

Arthroscopic Hip Surgery

Arthroscopic hip surgery performed in three studies involving 222 patients provided relevant data on intra-operative opioid consumption, opioid consumption at PACU, VAS pain score at PACU, and opioid consumption at 24 hours after surgery [25-27].

**Figure 3:**
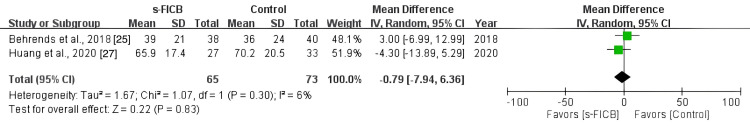
Forest plot comparing s-FICB to control for opioid consumption at 24 hours after arthroscopic hip surgery. s-FICB, suprainguinal fascia iliaca compartment block; CI, confidence interval; df, degrees of freedom; IV, inverse variance; SD, standard deviation

**Figure 4:**
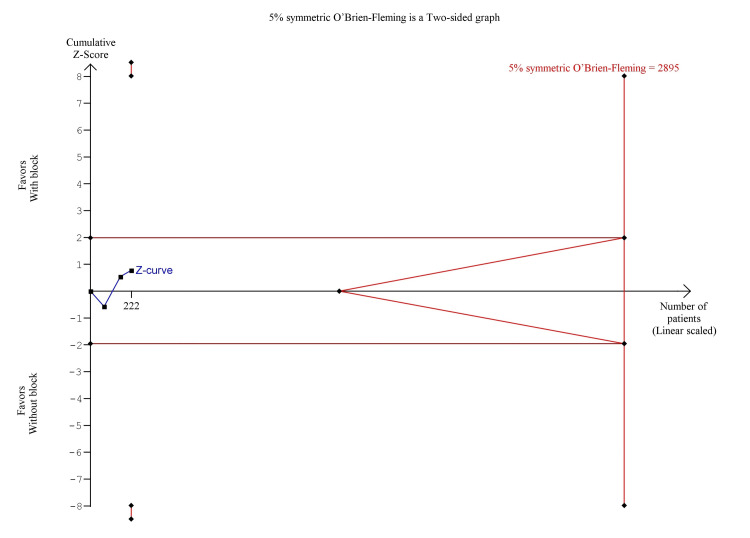
Trial sequence analysis comparing s-FICB to control for post-operative opioid consumption at 24 hours after arthroscopic hip surgery using the Biggerstaff-Tweedie model. s-FICB: suprainguinal fascia iliaca compartment block

As arthroscopic hip surgery is commonly a day surgery and patients are discharged on the same day, data collection after discharge is via telephone. The pooled analysis showed that s-FICB was associated with a non-significant difference in opioid consumption at 24 hours (MD, -0.79; 95% CI, -7.94 to 6.36; p = 0.83; I^2^ = 6%; Figure 3). On TSA for post-operative opioid consumption at 24 hours using the BT model, the cumulative Z-curve did not surprise both the traditional boundary for statistical significance and none of the trial sequential monitoring boundaries, demonstrating inconclusive results (Figure 4). The result remained the same when using the DL and SJ models. TSA on post-operative opioid consumption at PACU was not produced as there was too little information to produce the 5% symmetric O’Brien-Fleming boundary. The required sample size calculated was 2,895.

**Figure 5:**
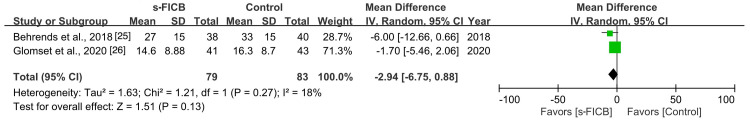
Forest plot comparing s-FICB to control for intra-operative opioid consumption during arthroscopic hip surgery. s-FICB, suprainguinal fascia iliaca compartment block; CI, confidence interval; df, degrees of freedom; IV, inverse variance; SD, standard deviation

**Figure 6:**
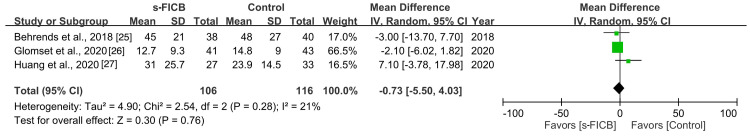
Forest plot comparing s-FICB to control for opioid consumption at post-anesthesia care unit (PACU) after arthroscopic hip surgery. s-FICB, suprainguinal fascia iliaca compartment block; CI, confidence interval; df, degrees of freedom; IV, inverse variance; SD, standard deviation

**Figure 7:**
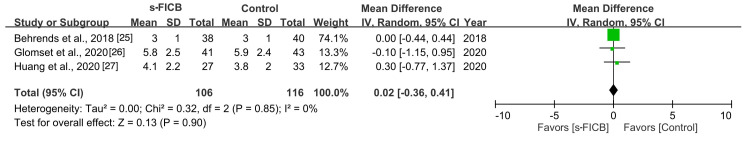
Forest plot comparing s-FICB to control for VAS pain score at post-anesthesia care unit (PACU) after arthroscopic hip surgery. s-FICB, suprainguinal fascia iliaca compartment block; CI, confidence interval; df, degrees of freedom; IV, inverse variance; SD, standard deviation; VAS, visual analog scale

The pooled analysis also did not reveal any significant difference in intra-operative opioid consumption (MD, -2.94; 95% CI, -6.75 to 0.88; p = 0.13; I^2^ = 18%; Figure 5), opioid consumption at PACU (MD, -0.73; 95% CI, -5.50 to 4.03; p = 0.76; I^2^ = 21%; Figure 6), and VAS pain score at PACU (MD, 0.02; 95% CI, -0.36 to 0.41; p = 0.90; I^2^ = 0%; Figure 7).

For opioid consumption beyond 24 hours, the included studies did not collect data from similar timepoints to perform a meta-analysis. In a study by Huang et al. (2020), opioid consumption and post-operative pain score were assessed for up to seven days and showed no statistically significant difference [27]. Glomset et al. (2020) followed up on their patients for up to three months, and similarly, they did not show a statistically significant difference between opioid consumption and post-operative pain score [26].

Hip and Femur Fracture Surgeries

Hip and femur fracture surgeries performed in three studies involving 149 patients provided relevant data on opioid consumption at 24 hours and VAS pain scores at 12 hours and 24 hours after surgery [28-30].

**Figure 8:**
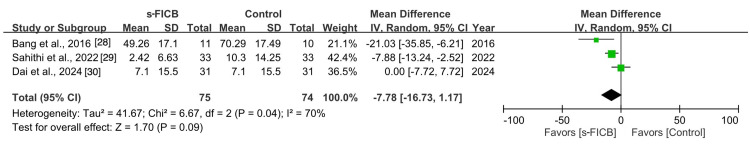
Forest plot comparing s-FICB to control for opioid consumption at 24 hours after hip fracture surgery. s-FICB, suprainguinal fascia iliaca compartment block; CI, confidence interval; df, degrees of freedom; IV, inverse variance; SD, standard deviation

**Figure 9:**
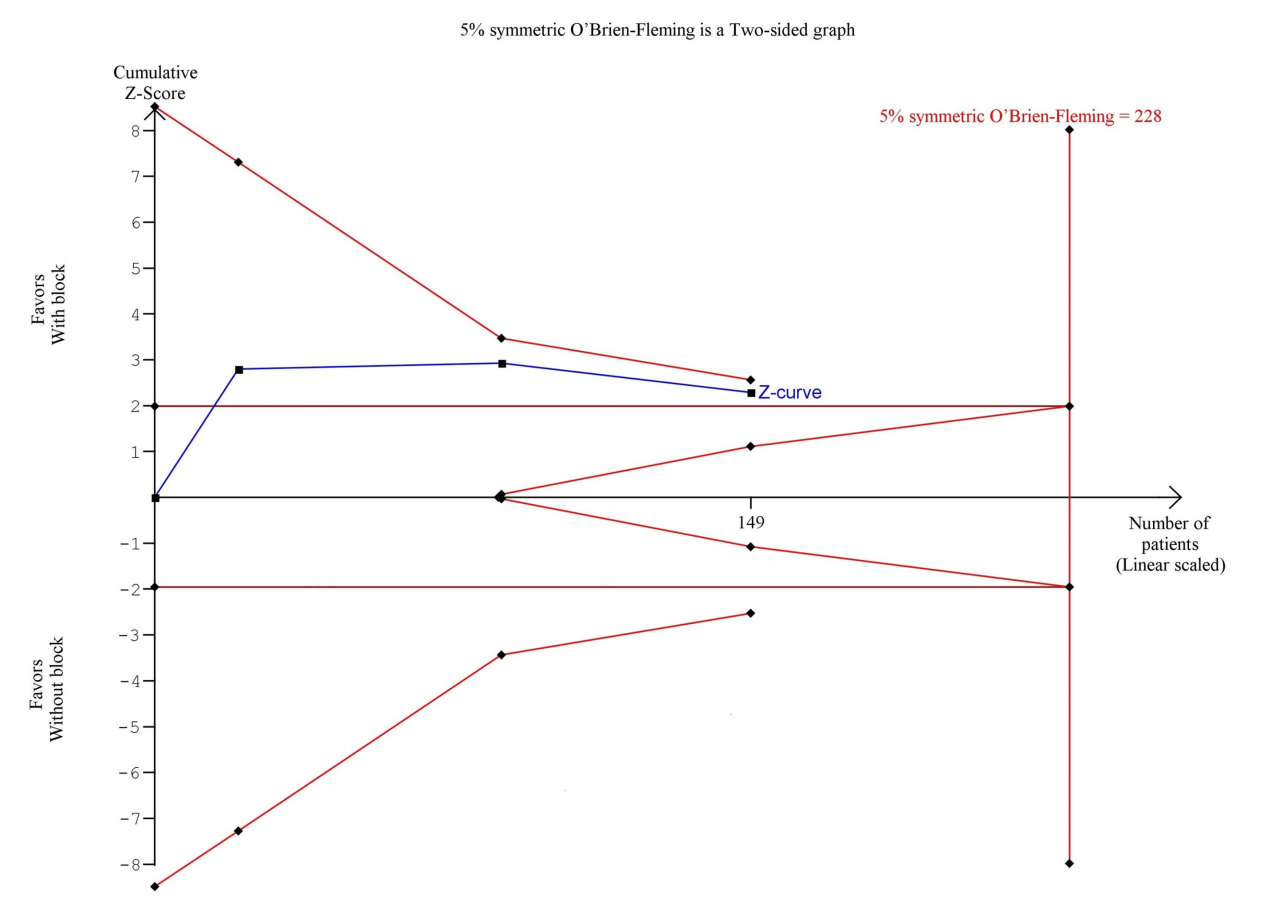
Trial sequence analysis comparing s-FICB to control for post-operative opioid consumption at 24 hours after hip fracture surgery using the Biggerstaff-Tweedie model. s-FICB: suprainguinal fascia iliaca compartment block

**Figure 10:**
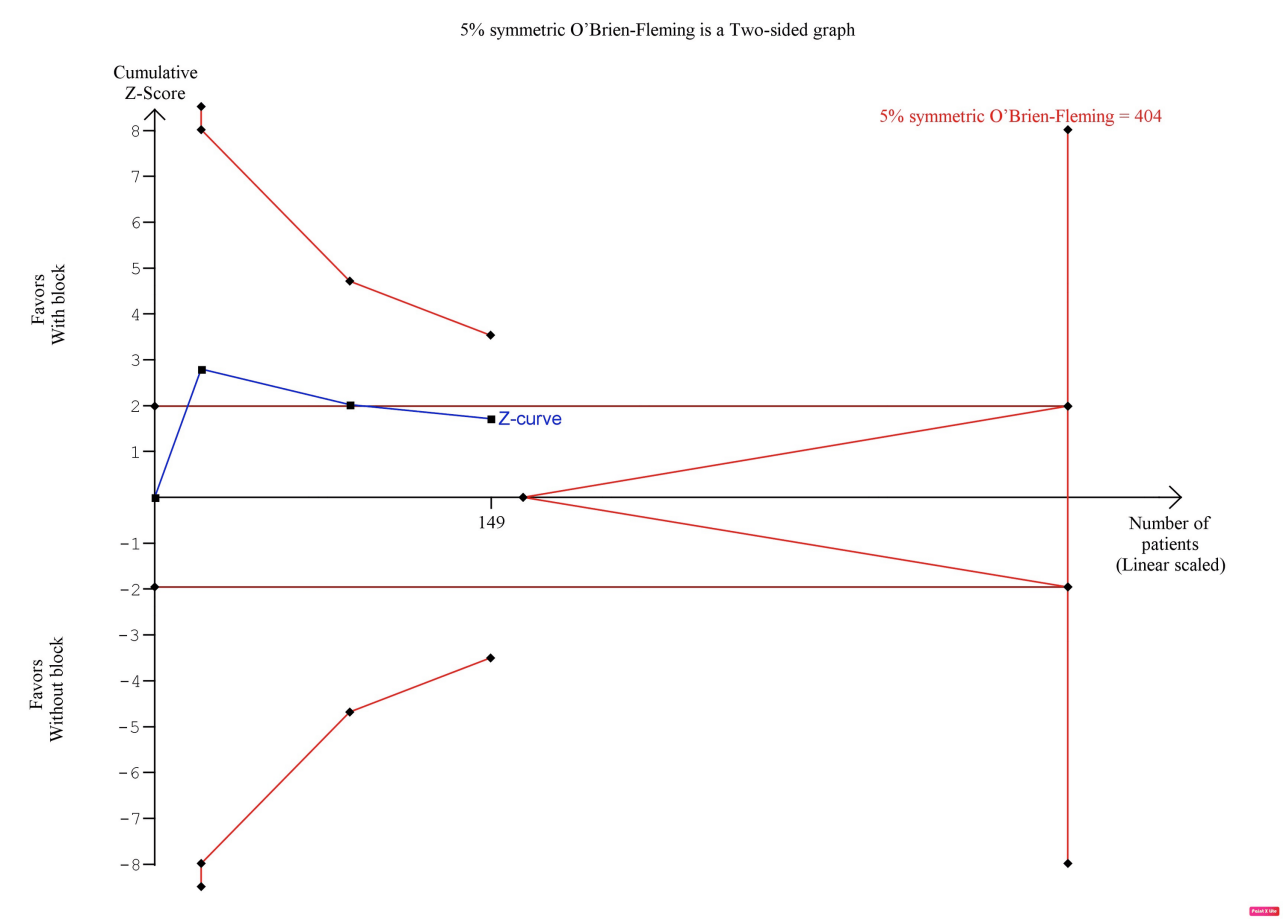
Trial sequence analysis comparing s-FICB to control for post-operative opioid consumption at 24 hours after hip fracture surgery using the DerSimonian-Laird model. s-FICB: suprainguinal fascia iliaca compartment block

**Figure 11:**
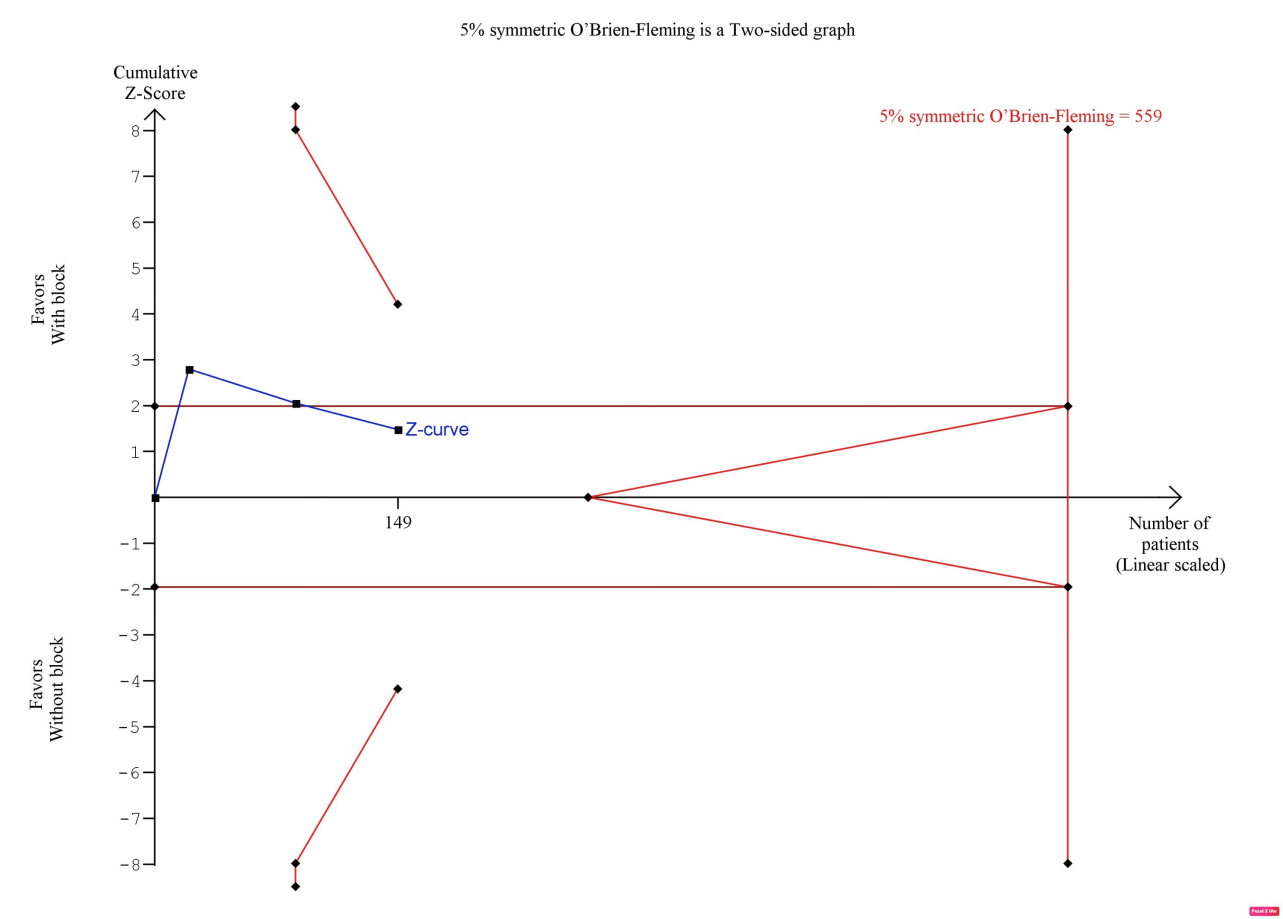
Trial sequence analysis comparing s-FICB to control for post-operative opioid consumption at 24 hours after hip fracture surgery using the Sidik-Jonkman model. s-FICB: suprainguinal fascia iliaca compartment block

The pooled analysis showed that s-FICB was associated with a non-significant difference in opioid consumption 24 hours after surgery (MD, -7.78; 95% CI, -16.73 to 1.17; p = 0.09; I^2^ = 70%; Figure 8). However, post-operative opioid consumption at 24 hours was inconclusive on TSA. In the BT model, although the cumulative Z-curve surpassed the traditional boundary for statistical significance, it did not reach the required information size (RIS), nor did it surpass any of the trial sequential monitoring boundaries (Figure 9). In the DJ and SJ models, the cumulative Z-curve did not surpass the traditional boundary as well (Figure 10 and Figure 11, respectively).

**Figure 12:**
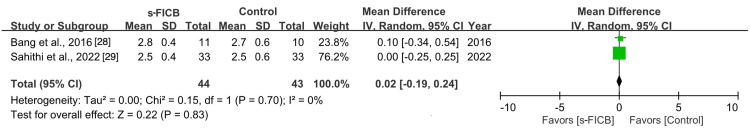
Forest plot comparing s-FICB to control for VAS pain score at 12 hours after hip fracture surgery. s-FICB, suprainguinal fascia iliaca compartment block; CI, confidence interval; df, degrees of freedom; IV, inverse variance; SD, standard deviation; VAS, visual analog scale

**Figure 13:**
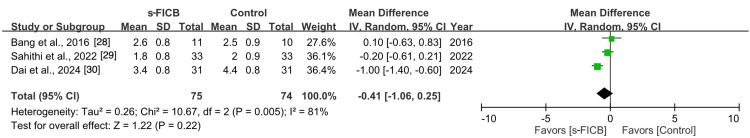
Forest plot comparing s-FICB to control for VAS pain score at 24 hours after hip fracture surgery. s-FICB, suprainguinal fascia iliaca compartment block; CI, confidence interval; df, degrees of freedom; IV, inverse variance; SD, standard deviation; VAS, visual analog scale

The pooled analysis also did not reveal any significant difference in post-operative pain score at 12 hours (MD, -0.02; 95% CI, -0.19 to 0.24; p = 0.83; I^2^ = 0%; Figure 12) and 24 hours after surgery (MD, -0.41; 95% CI, -1.06 to 0.25; p = 0.22; I^2^ = 81%; Figure 13).

Total Hip Arthroplasty

Total hip arthroplasty performed in five studies involving 483 patients provided relevant data on opioid consumption at 24 hours, opioid consumption at 48 hours, VAS pain score at 12 hours, VAS pain score at 24 hours, and VAS pain score at 48 hours after surgery [31-35].

**Figure 14:**
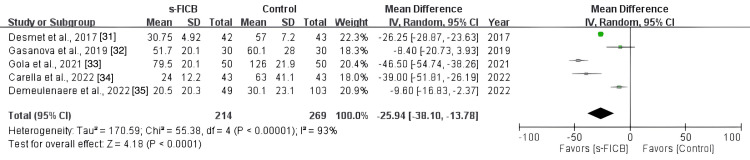
Forest plot comparing s-FICB to control for opioid consumption at 24 hours after total hip arthroplasty. s-FICB, suprainguinal fascia iliaca compartment block; CI, confidence interval; df, degrees of freedom; IV, inverse variance; SD, standard deviation

**Figure 15:**
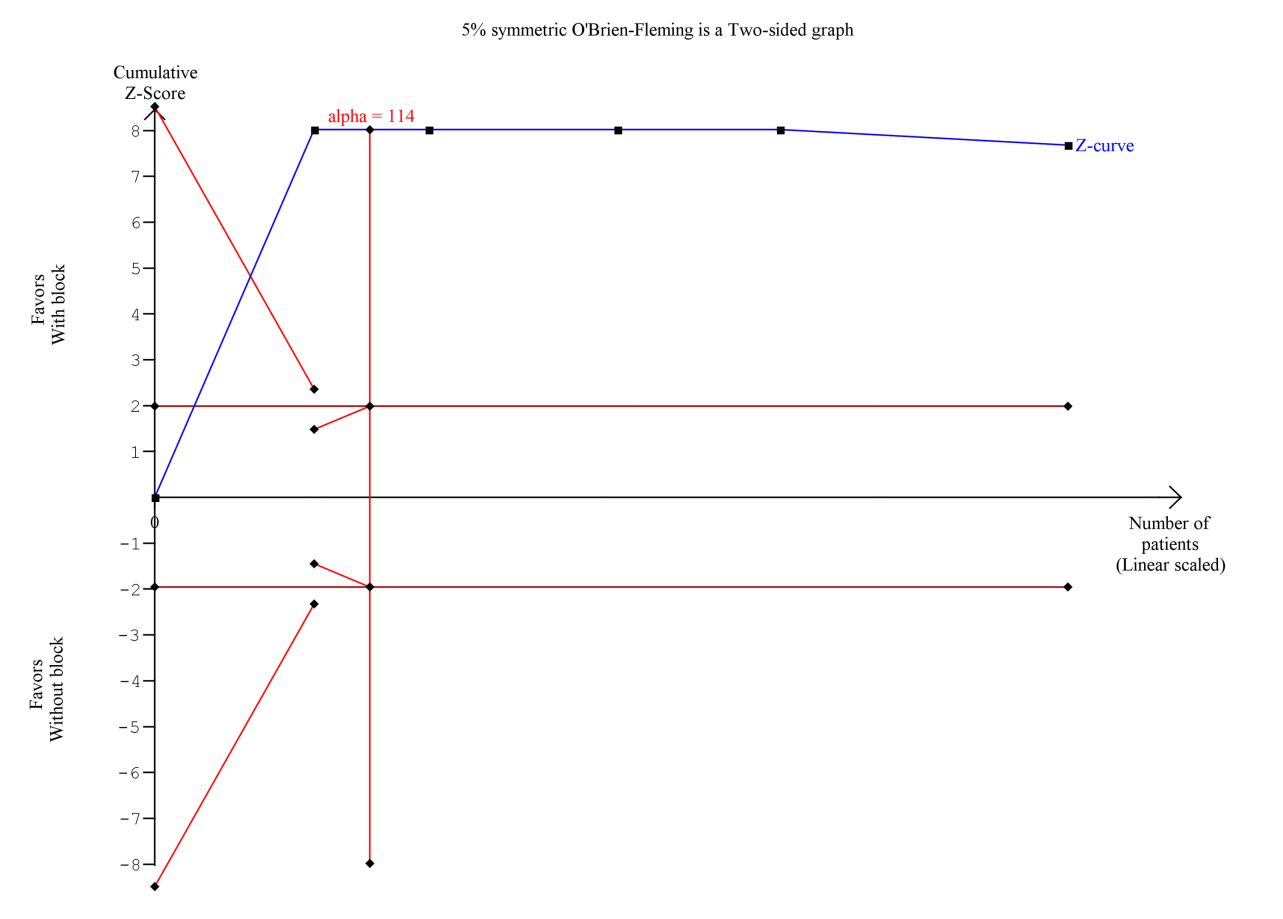
Trial sequence analysis comparing s-FICB to control for post-operative opioid consumption at 24 hours after total hip arthroplasty and hip fracture surgery using the Biggerstaff-Tweedie model. s-FICB: suprainguinal fascia iliaca compartment block

The pooled analysis showed that s-FICB was associated with a significant reduction in post-operative opioid consumption at 24 hours (MD, -25.94; 95% CI, -38.10 to -13.78; p < 0.00001; I^2^ = 93%; Figure 14). On TSA for post-operative opioid consumption at 24 hours using the BT model, the cumulative Z-curve surpasses the RIS and the trial sequential monitoring boundary for benefit (Figure 15). The result remained the same when using the DL and SJ models.

**Figure 16:**
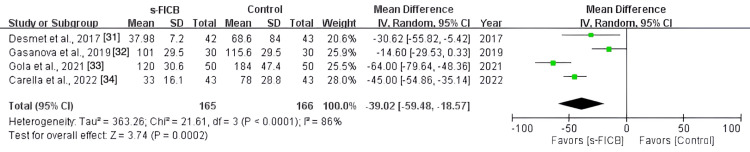
Forest plot comparing s-FICB to control for opioid consumption at 48 hours after total hip arthroplasty. s-FICB, suprainguinal fascia iliaca compartment block; CI, confidence interval; df, degrees of freedom; IV, inverse variance; SD, standard deviation

**Figure 17:**
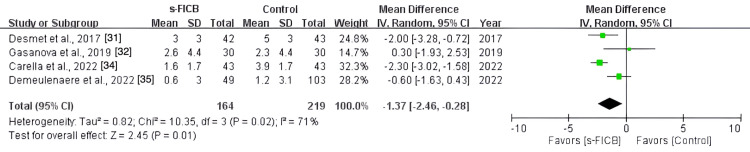
Forest plot comparing s-FICB to control for VAS pain score at PACU. s-FICB, suprainguinal fascia iliaca compartment block; CI, confidence interval; df, degrees of freedom; IV, inverse variance; SD, standard deviation; VAS, visual analog scale; PACU, post-anesthesia care unit

**Figure 18:**
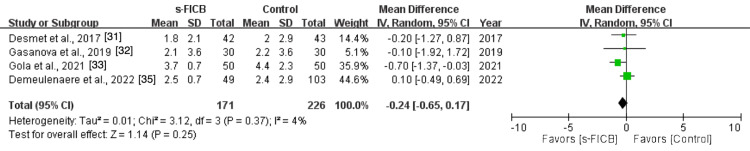
Forest plot comparing s-FICB to control for VAS pain score at 12 hours after total hip arthroplasty. s-FICB, suprainguinal fascia iliaca compartment block; CI, confidence interval; df, degrees of freedom; IV, inverse variance; SD, standard deviation; VAS, visual analog scale

**Figure 19:**
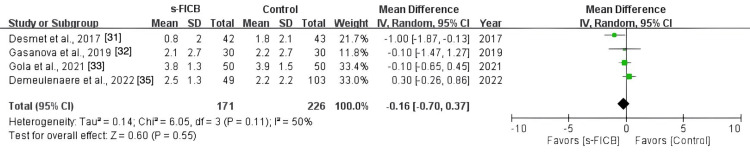
Forest plot comparing s-FICB to control for VAS pain score at 24 hours after total hip arthroplasty. s-FICB, suprainguinal fascia iliaca compartment block; CI, confidence interval; df, degrees of freedom; IV, inverse variance; SD, standard deviation; VAS, visual analog scale

**Figure 20:**
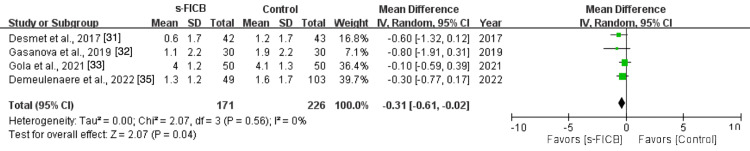
Forest plot comparing s-FICB to control for VAS pain score at 48 hours after total hip arthroplasty. s-FICB, suprainguinal fascia iliaca compartment block; CI, confidence interval; df, degrees of freedom; IV, inverse variance; SD, standard deviation; VAS, visual analog scale

The pooled analysis also showed a significant reduction in post-operative opioid consumption at 48 hours (MD, -39.02; 95% CI, -59.48 to -18.57; p < 0.0001; I^2^ = 86%; Figure 16) and a reduced pain score at PACU (MD, -1.37; 95% CI, -2.46 to -0.28; p = 0.01; I^2^ = 71%; Figure 17). However, there was no significant difference in post-operative pain score at 12 hours (MD, -0.24; 95% CI, -0.65 to 0.17; p = 0.37; I^2^ = 4%; Figure 18), 24 hours (MD, -0.16; 95% CI, -0.70 to 0.37; p = 0.11; I^2^ = 50%; Figure 19), and 48 hours (MD, -0.31; 95% CI, -0.61 to -0.02; p = 0.56; I^2^ = 0%; Figure 20).

Discussion

Elderly orthopedic patients are likely to have pre-existing medical comorbidities predisposing them to medical complications including, but not limited to, delirium, cardiopulmonary complications, venous thromboembolism, gastrointestinal tract bleeding, and urinary tract complications [36,37] and many of these complications are related to increased neurohormonal stress response from pain, prolonged immobility, and the use of centrally acting drugs including sedatives, anticholinergics, and opioids [37-40]. Regional anesthesia has been shown to reduce pain and quadricep spasm, opioid consumption, time to remobilization [41], the incidence of delirium in patients without pre-existing cognitive dysfunction [42,43], and the incidence of thromboembolism [44]. The Association of Anaesthetists’ guideline for the management of hip fractures 2020 recommended that patients with hip fractures should be provided single-shot nerve blocks in the emergency department (ED) and at the time of surgery [41]. Moreover, the European Society of Regional Anaesthesia and Pain Therapy’s PROSPECT recommendation 2024 suggested a single-shot femoral or FICB for pain management after hip fracture repair surgery [45]. However, multiple systematic reviews and meta-analyses on FICB have had mixed results for hip fracture surgery [10-13] and total hip arthroplasty surgery [14,15]. This may be due to the inclusion of using varying techniques of FICB, of which the older and inferior techniques may have diminished the overall effect size of the therapy. Our meta-analysis has only included studies in which the FICB was performed via the suprainguinal approach to observe the pooled effect of the more up-to-date s-FICB technique.

Our meta-analysis identified 11 RCTs that compared s-FICB to control in the patients undergoing hip surgeries. Hip surgeries identified include arthroscopic hip surgery (three studies), hip and femur fracture surgeries (three studies), and total hip arthroplasty (five studies). In patients who underwent arthroscopic hip surgery, the three included studies were identified and showed that s-FICB did not improve intra-operative and post-operative opioid consumption and post-operative pain score. However, TSA for post-operative opioid consumption was inconclusive. For those who underwent hip and femur fracture surgeries, the three included studies showed that s-FICB was associated with a non-significant difference in opioid consumption at 24 hours after surgery and post-operative pain score at 12 hours and 24 hours after surgery. However, the result of the TSA for post-operative opioid consumption at 24 hours was not definitive; therefore, additional research is necessary to draw conclusive outcomes. For total hip arthroplasty, the five included studies showed that s-FICB was associated with a significant reduction in post-operative opioid consumption at 24 and 48 hours. Moreover, the TSA for post-operative opioid consumption at 24 hours surpassed the RIS, suggesting adequate power to draw conclusion.

Our meta-analysis is limited by our strict inclusion criteria in only including studies that clearly stated the use of the s-FICB approach; therefore, we expected the number of included studies to be low. As a result, TSA was conducted to evaluate whether the sample size was sufficient to support our conclusions with adequate statistical power. The arthroscopic hip surgery group and the hip and femur fracture surgery group did not meet adequate sample size to make concrete conclusions. Moreover, the RIS is calculated to be 2,895 samples on TSA, suggesting a very low effect size, if any. For hip fracture surgery, the effect of s-FICB on post-operative opioid consumption remains inconclusive and warrants further evaluation. However, the total hip arthroplasty group manages to achieve conclusive results in the TSA, and this suggests clinical significance. This is in keeping with the meta-analysis performed by Cai et al. [10], Gao et al. [11], and Zhang et al. [12] but in disagreement with the more recent meta-analysis by Dai et al. [13].

There are multiple regional anesthesia options for post-operative analgesia after hip surgeries. The PENG block, an interfascial plane block that was first described by Girón-Arango et al. in 2018 as a motor-sparing regional anesthesia technique for total hip arthroplasties, has become popular in recent years. This aims to block the articular branches of the anterior hip joint supplied by femoral, obturator, and accessory obturator nerves and may be a viable alternative to s-FICB for hip surgery [46]. At the time of the literature search, there are four meta-analyses comparing the PENG block with FICB for hip arthroplasty surgeries, and this showed the superiority of the PENG block in reducing pain and minimizing opioid consumption; however, none of these meta-analyses have compared the PENG block with s-FICB specifically [47-50]. The effectiveness of the PENG block versus s-FICB to reduce pain and opioid consumption is mixed [47-51]. We would suggest that comparing the PENG block with the more effective s-FICB would be a better comparison for modern block techniques for hip surgeries.

## Conclusions

In conclusion, when performing FICB, a suprainguinal approach would be preferred as it provides superior performance when compared with the infrainguinal and landmark approaches. The number of studies on the use of s-FICB for hip surgery is limited. From the limited studies included in this systematic review, meta-analysis, and TSA, s-FICB is unlikely to show significant benefit in pain score and opioid requirement for arthroscopic hip surgery. However, for total hip arthroplasty, s-FICB showed a significant reduction in post-operative opioid consumption, and we would recommend performing s-FICB for patients undergoing total hip arthroplasty.

## References

[1] O'Reilly N, Desmet M, Kearns R (2019). Fascia iliaca compartment block. BJA Educ.

[2] Hsu YP, Hsu CW, Bai CH, Cheng SW, Chen C (2018). Fascia iliaca compartment block versus intravenous analgesic for positioning of femur fracture patients before a spinal block: a PRISMA-compliant meta-analysis. Medicine (Baltimore).

[3] Yun MJ, Kim YH, Han MK, Kim JH, Hwang JW, Do SH (2009). Analgesia before a spinal block for femoral neck fracture: fascia iliaca compartment block. Acta Anaesthesiol Scand.

[4] Dalens B, Vanneuville G, Tanguy A (1989). Comparison of the fascia iliaca compartment block with the 3-in-1 block in children. Anesth Analg.

[5] Dolan J, Williams A, Murney E, Smith M, Kenny GN (2008). Ultrasound guided fascia iliaca block: a comparison with the loss of resistance technique. Reg Anesth Pain Med.

[6] Hebbard P, Ivanusic J, Sha S (2011). Ultrasound-guided supra-inguinal fascia iliaca block: a cadaveric evaluation of a novel approach. Anaesthesia.

[7] Qian Y, Guo Z, Huang J (2020). Electromyographic comparison of the efficacy of ultrasound-guided suprainguinal and infrainguinal fascia iliaca compartment block for blockade of the obturator nerve in total knee arthroplasty: a prospective randomized controlled trial. Clin J Pain.

[8] Chen L, Shen Y, Liu S, Cao Y, Zhu Z (2021). Ultrasound-guided supra-inguinal fascia iliaca compartment block for older adults admitted to the emergency department with hip fracture: a randomized controlled, double-blind clinical trial. BMC Geriatr.

[9] Kumar K, Pandey RK, Bhalla AP (2015). Comparison of conventional infrainguinal versus modified proximal Suprainguinal approach of fascia iliaca compartment block for postoperative analgesia in total hip arthroplasty. A prospective randomized study. Acta Anaesthesiol Belg.

[10] Cai L, Song Y, Wang Z, She W, Luo X, Song Y (2019). The efficacy of fascia iliaca compartment block for pain control after hip arthroplasty: a meta-analysis. Int J Surg.

[11] Gao Y, Tan H, Sun R, Zhu J (2019). Fascia iliaca compartment block reduces pain and opioid consumption after total hip arthroplasty: a systematic review and meta-analysis. Int J Surg.

[12] Zhang XY, Ma JB (2019). The efficacy of fascia iliaca compartment block for pain control after total hip arthroplasty: a meta-analysis. J Orthop Surg Res.

[13] Dai W, Leng X, Hu X, Cheng J, Ao Y (2021). The effect of fascia iliaca block on postoperative pain and analgesic consumption for patients undergoing primary total hip arthroplasty: a meta-analysis of randomized controlled trials. J Orthop Surg Res.

[14] Hong HK, Ma Y (2019). The efficacy of fascia iliaca compartment block for pain control after hip fracture: a meta-analysis. Medicine (Baltimore).

[15] Baker HP, Portney DA, Schroedl LM, Strelzow JA, Hynes K, Dillman DB (2022). The effect of fascia iliaca compartment blockade on mortality in patients with hip fractures: systematic review and meta-analysis of randomized controlled trials. J Am Acad Orthop Surg.

[16] Moher D, Shamseer L, Clarke M (2015). Preferred reporting items for systematic review and meta-analysis protocols (PRISMA-P) 2015 statement. Syst Rev.

[17] Guyatt GH, Oxman AD, Vist GE, Kunz R, Falck-Ytter Y, Alonso-Coello P, Schünemann HJ (2008). GRADE: an emerging consensus on rating quality of evidence and strength of recommendations. BMJ.

[18] (2023). Opioid dose equivalence calculation table. https://www.anzca.edu.au/getattachment/6892fb13-47fc-446b-a7a2-11cdfe1c9902/PS01-Opioid-Dose-Equivalence-Calculation-Table.

[19] Hozo SP, Djulbegovic B, Hozo I (2005). Estimating the mean and variance from the median, range, and the size of a sample. BMC Med Res Methodol.

[20] Furukawa TA, Barbui C, Cipriani A, Brambilla P, Watanabe N (2006). Imputing missing standard deviations in meta-analyses can provide accurate results. J Clin Epidemiol.

[21] Luo D, Wan X, Liu J, Tong T (2018). Optimally estimating the sample mean from the sample size, median, mid-range, and/or mid-quartile range. Stat Methods Med Res.

[22] Shi J, Luo D, Wan X, Liu Y, Liu J, Bian Z, Tong T (2023). Detecting the skewness of data from the five-number summary and its application in meta-analysis. Stat Methods Med Res.

[23] Shi J, Luo D, Weng H, Zeng XT, Lin L, Chu H, Tong T (2020). Optimally estimating the sample standard deviation from the five-number summary. Res Synth Methods.

[24] Wan X, Wang W, Liu J, Tong T (2014). Estimating the sample mean and standard deviation from the sample size, median, range and/or interquartile range. BMC Med Res Methodol.

[25] Behrends M, Yap EN, Zhang AL, Kolodzie K, Kinjo S, Harbell MW, Aleshi P (2018). Preoperative fascia iliaca block does not improve analgesia after arthroscopic hip surgery, but causes quadriceps muscles weakness: a randomized, double-blind trial. Anesthesiology.

[26] Glomset JL, Kim E, Tokish JM, Renfro SD, Seckel TB, Adams KJ, Folk J (2020). Reduction of postoperative hip arthroscopy pain with an ultrasound-guided fascia iliaca block: a prospective randomized controlled trial. Am J Sports Med.

[27] Huang MJ, Wages JJ, Henry AC, Epperson JM (2020). Should preoperative fascia iliaca block be used for hip arthroscopic labral repair and femoroacetabular impingement treatment? A prospective single blinded randomized study. Arthroscopy.

[28] Bang S, Chung J, Jeong J, Bak H, Kim D (2016). Efficacy of ultrasound-guided fascia iliaca compartment block after hip hemiarthroplasty: a prospective, randomized trial. Medicine (Baltimore).

[29] Sahithi TO, Venkatraman RA, Swetharamani CK, Karthik K (2022). Evaluation of ultrasound-guided pre-emptive fascia iliaca compartment block for postoperative analgesia in femur and hip fracture surgeries: a randomised controlled trial. J Clin Diagnostic Res.

[30] Dai X, Xing D, Luo J, Yang Y, Zhai J, Tang T, Yang W (2024). Fascia iliaca compartment block mitigates the fluctuations in heart rate variability and reduces pain with opioid consumption in elderly individuals with hip fractures: a randomized controlled trial. Heliyon.

[31] Desmet M, Vermeylen K, Van Herreweghe I (2017). A longitudinal supra-inguinal fascia iliaca compartment block reduces morphine consumption after total hip arthroplasty. Reg Anesth Pain Med.

[32] Gasanova I, Alexander JC, Estrera K, Wells J, Sunna M, Minhajuddin A, Joshi GP (2019). Ultrasound-guided suprainguinal fascia iliaca compartment block versus periarticular infiltration for pain management after total hip arthroplasty: a randomized controlled trial. Reg Anesth Pain Med.

[33] Gola W, Bialka S, Owczarek AJ, Misiolek H (2021). Effectiveness of fascia iliaca compartment block after elective total hip replacement: a prospective, randomized, controlled study. Int J Environ Res Public Health.

[34] Carella M, Beck F, Piette N, Denys S, Kurth W, Lecoq JP, Bonhomme VL (2022). Effect of suprainguinal fascia iliaca compartment block on postoperative opioid consumption and functional recovery in posterolateral-approached total hip arthroplasty: a single-blind randomized controlled trial. Reg Anesth Pain Med.

[35] Demeulenaere M, Janssens GP, van Beek N, Cannaerts N, Tengrootenhuysen MM (2022). Optimizing rapid recovery after anterior hip arthroplasty surgery: a comparative study of fascia iliaca compartment block and local infiltration analgesia. J Arthroplasty.

[36] Potter JF (2004). The older orthopaedic patient: general considerations. Clin Orthop Relat Res.

[37] Carpintero P, Caeiro JR, Carpintero R, Morales A, Silva S, Mesa M (2014). Complications of hip fractures: a review. World J Orthop.

[38] Morrison RS, Magaziner J, Gilbert M (2003). Relationship between pain and opioid analgesics on the development of delirium following hip fracture. J Gerontol A Biol Sci Med Sci.

[39] Morrison RS, Magaziner J, McLaughlin MA, Orosz G, Silberzweig SB, Koval KJ, Siu AL (2003). The impact of post-operative pain on outcomes following hip fracture. Pain.

[40] Summers S, Grau L, Massel D, Rosas S, Ong A, Hernandez VH (2018). Opioid use disorders are associated with perioperative morbidity and mortality in the hip fracture population. J Orthop Trauma.

[41] Griffiths R, Babu S, Dixon P (2021). Guideline for the management of hip fractures 2020: guideline by the Association of Anaesthetists. Anaesthesia.

[42] Kim CH, Yang JY, Min CH, Shon HC, Kim JW, Lim EJ (2022). The effect of regional nerve block on perioperative delirium in hip fracture surgery for the elderly: a systematic review and meta-analysis of randomized controlled trials. Orthop Traumatol Surg Res.

[43] Jia B, Tang Y, Wei C, Zhao G, Li X, Shi Y (2023). Peripheral nerve block and peri-operative neurocognitive disorders in older patients with hip fractures: a systematic review with meta-analysis. Geriatr Orthop Surg Rehabil.

[44] Ho HH, Lau TW, Leung F, Tse HF, Siu CW (2010). Peri-operative management of anti-platelet agents and anti-thrombotic agents in geriatric patients undergoing semi-urgent hip fracture surgery. Osteoporos Int.

[45] Pissens S, Cavens L, Joshi GP (2024). Pain management after hip fracture repair surgery: a systematic review and procedure-specific postoperative pain management (PROSPECT) recommendations. Acta Anaesthesiol Belg.

[46] Girón-Arango L, Peng PW, Chin KJ, Brull R, Perlas A (2018). Pericapsular nerve group (PENG) block for hip fracture. Reg Anesth Pain Med.

[47] Aliste J, Layera S, Bravo D (2021). Randomized comparison between pericapsular nerve group (PENG) block and suprainguinal fascia iliaca block for total hip arthroplasty. Reg Anesth Pain Med.

[48] Carella M, Beck F, Piette N, Denys S, Lecoq JP, Bonhomme VL (2023). Comparison between supra-inguinal fascia iliaca and pericapsular nerve group blocks on postoperative pain and functional recovery after total hip arthroplasty: a noninferiority randomised clinical trial. Eur J Anaesthesiol.

[49] Duan L, Zhang L, Shi CG (2023). Comparison of continuous pericapsular nerve group (PENG) block versus continuous fascia iliaca compartment block on pain management and quadriceps muscle strength after total hip arthroplasty: a prospective, randomized controlled study. BMC Anesthesiol.

[50] Noaman SS, Abdallah ES, Elsawy SM, Abd El-Radi M, Kamel MM (2023). The efficacy of pericapsular nerve group block versus facia iliaca block on immediate postoperative pain and opioid consumption after hip arthroscopy randomized trial. Pain Physician.

[51] Liang L, Zhang C, Dai W, He K (2023). Comparison between pericapsular nerve group (PENG) block with lateral femoral cutaneous nerve block and supra-inguinal fascia iliaca compartment block (S-FICB) for total hip arthroplasty: a randomized controlled trial. J Anesth.

